# Self-rated health of population in southern China: association with socio-demographic characteristics measured with multiple-item self-rated health measurement scale

**DOI:** 10.1186/1471-2458-10-393

**Published:** 2010-07-03

**Authors:** Jun Xu, Jinhua Zhang, Liyi Feng, Jincai Qiu

**Affiliations:** 1 Department of Pharmacology, Nanfang Hospital, Southern Medical University, Guangzhou, Guangdong Province, PR China; 2 School of Public Health and Tropical Medicine, Southern Medical University, Guangzhou, Guangdong Province, PR China

## Abstract

**Background:**

Self-rated health (SRH) status has been increasingly acknowledged as a valid and appropriate indicator of public health and chronic morbidity. However, limited research was conducted in China due to the different culture and socioeconomic situations. The aim of this study is to assess the SRH status of the population in Southern China using multiple-item SRH measurement scale (SRHMS). Socio-demographic characteristics including sex, age, marital status, education, and income are considered variable in this survey.

**Methods:**

A cross-sectional survey was conducted in a total of 8400 community residents of 14 years old and over in Southern China. SRH status was measured using SRHMS with a stratified sampling approach, and compared between different subgroups with t-test and one-way analysis of variance (ANOVA).

**Results:**

Totally 8400 subjects were recruited in this study and 80.96% (6801) responded to the survey. The mean score for SRHMS dimensions ranged from 66.16 ± 20.65 (mean ± sd) for positive emotion (M2) to 92.14 ± 14.06 for daily physical activities (B2). Results showed that SRHMS scores for women, elderly men, low education level, low income, divorced, separated or widowed and suburban residents in Southern China were significantly lower than other subgroups (P < 0.05).

**Conclusions:**

In this study, using SRHMS we assessed the association of SRH with socio-demographic characteristics including sex, age, marital status, education, and income in Southern China. The performance of the questionnaire in the large scale survey is satisfactory and provides a large picture of SRH status in Southern China. Our results indicate that women, elderly men, low education level, low income, divorced, separated or widowed and suburban residents in Southern China suffer from relatively poor SRH status.

## Background

Based on the World Health Organization (WHO) definition, health is a state of complete physical, mental and social well-being and not merely the absence of disease or infirmity[1]. This definition has not been amended since 1948. Over the past decades, the Chinese have enjoyed a rapidly declining mortality rates and a long life expectancy at all ages[2,3]. Today the primary focus of medical care has shifted from infections diseases to chronic diseases[4,5]. However, over the past few years, we have observed an increase in health service attendance due to unspecific somatization condition and chronic diseases. People in China no longer consent to the absence of diseases and dysfunction. They are pursuing more comfortable life and overall well-being[6]. In respect of this, more cost-effective tools should be developed for the health surveillance.

In the public health research and practice, self-rated health (SRH) is generally considered a valuable source of information on subjective health status and is widely adopted due to its simplicity to collect[7]. SRH has been recognized as a valid and appropriate indicator of service need and intervention outcomes[8,9]. In developed countries, SRH has been shown to be an independent predictor of mortality[10-13] and has a high reliability, validity and predictive power for a variety of illness and conditions[14]. Currently, SRH has been recommended for population health monitoring by WHO, the Centers for Disease Control and Prevention in the United States, and the European Union Commission[12,13,15,16]. Its determinants have also been well documented in developed world[17-20] For instance, Simonsen et al[21]. have revealed the relationship between weight changes and SRH. However, SRH and its determinants are not investigated adequately in China. Mechanisms underlying routinely monitoring health status of populations at national and local level are currently missing. Studies have emphasized that health measurement may be influenced by the cultural and socioeconomic situation in different countries[22-24]. Thus, it is critical for China to develop appropriate scales, which are suitable for the Chinese culture and socioeconomic situation. These scales will help understand the factors contributing to SRH and improvement of the primary health care services.

When measuring the SRH status, single-item measures are popular because they are quick and easy to administer in a large-scale survey. Studies have also shown that a single-item measure of overall health is sufficiently sensitive to reveal relationships with a number of predictor variables[25]. However, it has been shown that single-item SRH as a dependent variable is an unspecific indicator of health[26,27]. According to WHO's definition, health can be viewed as a multidimensional concept, which may include physical health, mental health and social well-being. Thus, it is impractical for a single-item question to capture the characteristic of health status adequately. Furthermore, the single-item scale may not give enough information to reveal population's health status in primary care research[27]. If primary research objective focuses on the prevention and control of chronic diseases and improving the public health care services, managers and policy makers are unlikely willing to make significant management or policy decisions based upon data from a single-item measure[27,28].

Given this background, Xu et al. developed a multiple-item SRH measurement scale (SRHMS)[29-35]. Social, historical, and cultural factors were taken into account in the SRHMS scale. It seems suitable for hospitalized and general population, and has a high reliability, validity and sensitive to the alteration of the health status[29,31,32,35].

However, information about the performance of such instruments in different population groups is scarce and formal comparisons are lacking. In this study, we explored globally the performance of SRHMS questionnaires in different socio-demographic characteristics such as gender, age, education, and so on.

In general, the socio-demographic characteristics include age, sex, marital status, living arrangements, household composition, education, income, social class, ethnicity, and occupational class. Studies have shown that these factors are closely related to the health status. Social class is typically used in sociology as a central theoretical concept indicating the individual's location in the social stratification system and access to material resources, influence and information. Social class is thought to affect health and mortality in many ways: by influencing attitudes, beliefs and values people use to make life-course choices and by influencing life-course opportunities. In China, however, there is no clear definition of social class and most people are not clear which social class they belong to. People prefer to classify themselves into a sub-class based on the income level such as low income, medium-income and high-income. Therefore, in this study we didn't include social class as a variable. Ethnic background has been identified as one of the factors that influence health and mortality. A number of studies have shown the influence of ethnic and cultural background on health and have pointed to the facts that disease and mortality are functions of social culture as well as of class, and that different diseases prevail in different cultures. Residencies in Southern China predominantly belong to a single race and have Chinese traditional Confucian culture. Therefore, we didn't include ethnic/culture background as variable either.

## Methods

### Study population and data collection

This was a cross-sectional survey of a random sample of southern Chinese selected from the general population in five Southern China cities. Southern China is the most densely populated region in China. The sampling method was based on a stratified random sampling approach. Five cities (Guangzhou, Shenzhen, Maoming, Shaoguan, and Shantou) were selected following the sequence of the district-block-residential area. These five cities represent typical level in respect of the city scale and geographical distribution. Shenzhen and Guangzhou are typical great metropolises. Maoming and Shantou are representatives of the medium-sized cities. Shaoguan is a representative of a small city in remote mountain areas. Overall, these five cities represent the characteristics of different types including a new city with immigrants, a city of hundreds of years history, a coastal city and an inland city. Therefore, the survey of residencies from these cities could well represent the SRH status of the populations in Southern China. Streets or villages were randomly selected in five cities. Then participants were randomly selected from the eligible candidates listed in residential registration record. All the participants must be aged 14 years or above. Age was categorized as <24 years, 25-34 years, 35-44 years, 45-54 years, 55-64 years, 65-74 years, and 75-years intervals. Proportionate allocation sampling was used to identify a sampling fraction for each districts, age segment and gender. But we have adjusted the sample size of the <24 years' and 75 years' groups according to the proportion of Chinese population. The sample size of <24 years, 25-34 years, 35-44 years, 45-54 years, 55-64 years, 65-74 years, 75 years were 1400, 1200, 1200, 1200, 1200, 1200 and 1000 respectively. The proportion of male and female were 50% in each district and age segment sample.

All the respondents gave written informed consent to all assessments reported and the study was approved by Nanfang hospital ethics committee.

### Questionnaire

The questionnaire included general information and the revision of SRHMS. General information was collected on age, sex, resident region, nationality, marital status, educational level, current job, and personal monthly income. The revision of SRHMS[31-34] consisted of 48 items. These items were divided into 9 dimensions: physical symptom and organic function (B1), daily physical activities (B2), physical mobility (B3), psychosocial symptom and negative emotion (M1), positive emotion (M2), cognitive function (M3), role activity and social adaptability (S1), social resource and social contact (S2), and social support (S3). The nine dimensions were also categorized into 3 subscales: physical health, mental health and social health. The summarized scores of SHRMS and each subscale were represented by SCZT, BZT, MZT and SZT respectively. Each item is rated on a horizontal line, 10 cm in length, on which people rate their score. Raw scores can range from 0 to 10 cm, including fractions of a centimeter. Each of the 48 items has a maximum possible score of 10 and a minimum possible score of 0. Four items are not counted into the total score. Thus the maximum possible score is 440[32]. Item 4, 5, 7, 24, 25, 26, 27, 28, 29, 30 are scored inversely so that a higher number indicates impairment (i.e.1.5 = 10-8.5, 9 = 10-1, and2 = 10-8, etc)[32]. SRHMS is a self administered instrument that takes approximately 15 minutes to complete. The directions are simple and the scoring is self-explanatory.

### Field Work

Twelve interviewers were trained to collect the data. All participants were interviewed at their home or in local resident committees. Participants were asked to fill the questionnaire to assess their SRH. The interviewers only provided necessary explanation without any inducement on the unclear questions.

### Statistics

The raw score of each nine SRHMS dimensions was derived by summing the item scores, and converted to a value for the dimension from 0 (worst possible health status measured by the questionnaire) to 100 (best possible health status). The raw score was then re-calculated across the dimension as follow:

Statistical analysis was carried out by using SPSS for windows (Southern Medical University, China. release17^®^). The statistical description of the clinical and socio-demographic variables was performed by using frequencies, percentages, means, and standard deviations. To examine the associations between the participants' characteristics and their SRH, univariate analysis including t-tests and one-way analysis of variance (ANOVA) were performed. Two-sided p-values less than 0.05 were considered significant.

## Results

### Demographic characteristics of the participants

Totally 8400 participants received the interview. Among them, 1599 failed to complete their SRHMS assessment. The other 6801 participants were eligible for data analyses. The response rate was 80.96%.

As shown in Table 1, 3362 (49.43%) participants were females, 3413 (50.18%) were males. The mean (sd) age of the participants was 44.93 (19.45). Most of the participants (63.27%) were married. The proportions of the participants with low, medium and high education were 2266 (33.32%), 2095 (30.80%), 1791 (26.33%) respectively. Most of the participants (71.77%) lived in the urban residence.

**Table 1:** Frequency distribution of the participant's demographical characteristics (n = 6801)

	Characteristics	Number	Percent (%)
Sex			
	Female	3362	49.43
	Male	3413	50.18
	Information missing	26	0.38
Age			
	14~	1339	19.69
	25~	1059	15.57
	35~	1002	14.73
	45~	1094	16.09
	55~	911	13.40
	65~	878	12.91
	75~	505	7.43
	Information missing	13	0.19
Education^a^			
	Low education	2266	33.32
	Medium education	2095	30.80
	High education	1791	26.33
	Illiterate or other	503	7.40
	Information missing	146	2.15
Marital Status			
	Married	4303	63.27
	Non-married	1672	24.58
	Divorced/separated/widowed	571	8.40
	Information missing	255	3.75
Residence			
	Urban	4881	71.77
	Rural	1012	14.88
	Suburban	655	9.63
	Information missing	253	3.72
City			
	Guangzhou	1323	19.45
	Maoming	1335	19.63
	Shantou	1269	18.66
	Shaoguan	1297	19.07
	Shengzhen	1577	23.19
	Information missing	0	0.00
Income(RMB)^b^			
	0~	1471	21.63
	500~	1288	18.94
	1000~	1440	21.17
	2000~	1057	15.54
	Information missing	1545	22.07

Note: ^a^Low education: Primary school and junior high school; Medium education: high school, technical secondary school, vocational school; High education: some college, junior college, college or higher. ^b^Income: after-tax income of a full-time work, which includes basic salary and bonus (after taxes) but not public assistance/benefits, help from relatives, alimony, and the income of a part-time job.

### Scores of SRHMS

The mean (sd) of the SRHMS summary raw scores was 326.80 (52.94). The mean (sd) of the physical, mental and social health subscale raw summary scores were 134.48 (22.38), 105.60 (22.29) and 86.72 (18.35) respectively in this study. The SRH transformed scores measured by SRHMS were presented in Table 2. The mean score for the SRHMS dimensions ranged from 66.16 (20.65) for positive emotion (M2) to 92.14 (14.06) for daily physical activities (B2). Generally, people in Southern China have a relatively higher SRHMS score in daily physical activities dimension (B2 = 92.14) and physical mobility dimension (B3 = 84.16).

**Table 2:** Scores of SRHMS and mean scores of SRH of all the participants (n = 6801)

Scales*	Maximum possible raw score	Maximum possible transformed score	Means	SD
B1	70.00	100.00	66.18	15.30
B2	50.00	100.00	92.14	14.06
B3	50.00	100.00	84.16	19.00
BZT	170.00	100.00	79.10	13.17
M1	50.00	100.00	77.20	16.46
M2	70.00	100.00	66.16	20.65
M3	30.00	100.00	68.96	18.45
MZT	150.00	100.00	70.40	14.86
S1	40.00	100.00	77.25	15.69
S2	50.00	100.00	71.92	19.33
S3	30.00	100.00	66.21	19.73
SZT	120.00	100.00	72.27	15.29
ZCZT	440.00	100.00	74.28	12.03

*Note*: B1: Physical symptom and organic function; B2: Daily physical activities; B3: Physical mobility; BZT: Total score of physical health subscale; M1: Psychosocial symptom and negative emotion; M2: Positive emotion; M3: Cognitive function: MZT: Total score of psychosocial health subscale; S1: Role activity and social adaptability; S2: Social resource and social contact; S3: Social support; SZT: Total score of social health subscale; ZCZT: Total score of the SRHMS

### Association between participants' socio-demographic characteristics and SRH

The association between the participants' socio-demographic characteristics and their SRH scores were presented in Table 3,4,5,6,7 and 8. In this study, the socio-demographic characteristics included sex, age, education, region, marital status and personal monthly income.

**Table 3:** Comparison between male and female in each dimension of SRHMS (n = 6801)

Dimension	Sex		*t*	*P*
			
	Male	Female		
**B1**	67.52 ± 15.26	64.83 ± 15.23	7.268	0.000
**B21**	92.47 ± 13.49	91.78 ± 14.64	2.201	0.043
**B3**	85.85 ± 18.40	82.40 ± 19.46	7.496	0.000
**BZT**	80.25 ± 12.86	77.92 ± 13.39	7.292	0.000
**M1**	77.83 ± 16.06	76.55 ± 16.84	3.183	0.001
**M2**	67.28 ± 20.47	65.04 ± 20.77	4.479	0.000
**M3**	70.47 ± 18.35	67.40 ± 18.45	6.859	0.000
**MZT**	71.43 ± 14.68	69.35 ± 14.96	5.79	0.000
**S1**	78.28 ± 15.38	76.21 ± 15.92	5.449	0.000
**S2**	72.77 ± 19.22	71.03 ± 19.43	3.701	0.000
**S3**	65.83 ± 19.79	66.58 ± 19.66	1.563	0.118
**SZT**	72.87 ± 15.12	71.64 ± 15.44	3.306	0.001
**ZCZT**	75.23 ± 11.83	73.29 ± 12.16	6.67	0.000

**Table 4:** Comparison among different age group in each dimension of SRHMS (n = 6801)

Dimension	Age							*F*	*P*
			
	**14**~	25~	35~	45~1	55~	65~	75~		
**B1**	70.51 ± 13.76	70.97 ± 14.77	69.69 ± 14.47	65.24 ± 13.79	64.35 ± 14.49	60.05 ± 15.07	53.61 ± 15.40	142.916	0.000
**B2**	94.83 ± 11.74	95.90 ± 8.51	94.65 ± 10.04	94.14 ± 10.32	91.67 ± 13.69	86.86 ± 17.18	77.80 ± 23.23	156.912	0.000
**B3**	93.12 ± 11.11	92.55 ± 9.90	89.93 ± 12.02	85.91 ± 14.71	80.52 ± 18.05	70.90 ± 21.90	56.95 ± 25.34	507.55	0.000
**BZT**	84.31 ± 9.24	84.65 ± 8.76	82.99 ± 9.50	79.82 ± 9.97	77.14 ± 12.24	71.13 ± 14.89	61.71 ± 17.56	380.696	0.000
**M1**	79.24 ± 15.02	79.28 ± 15.00	78.86 ± 15.22	77.36 ± 16.58	75.84 ± 16.55	74.05 ± 17.94	71.75 ± 19.62	24.077	0.000
**M2**	65.99 ± 19.30	66.50 ± 21.26	66.82 ± 21.75	65.29 ± 20.59	66.12 ± 21.29	66.43 ± 20.90	66.22 ± 19.04	0.581	0.746
**M3**	73.04 ± 16.14	75.60 ± 15.39	74.17 ± 15.85	68.83 ± 17.21	66.57 ± 18.02	61.22 ± 19.41	51.96 ± 20.45	168.61	0.000
**MZT**	71.81 ± 13.92	72.58 ± 14.49	72.30 ± 14.28	70.03 ± 14.47	69.45 ± 15.04	67.93 ± 15.84	65.21 ± 15.74	24.123	0.000
**S1**	79.55 ± 13.44	82.32 ± 12.86	81.18 ± 13.24	78.66 ± 14.62	74.53 ± 16.38	71.29 ± 17.32	65.01 ± 18.29	124.579	0.000
**S2**	75.41 ± 17.79	74.76 ± 17.98	74.10 ± 17.33	73.77 ± 18.00	70.67 ± 19.03	66.53 ± 21.09	59.85 ± 22.88	62.945	0.000
**S3**	67.73 ± 18.65	67.37 ± 20.05	66.79 ± 19.21	67.02 ± 19.21	64.72 ± 19.62	64.59 ± 20.79	62.27 ± 21.41	7.651	0.000
**SZT**	74.87 ± 13.52	75.43 ± 14.05	74.63 ± 13.33	73.71 ± 14.39	70.47 ± 15.20	67.63 ± 17.09	62.17 ± 17.92	76.71	0.000
**ZCZT**	77.48 ± 9.81	78.02 ± 10.08	77.07 ± 10.12	74.81 ± 10.56	72.70 ± 11.74	69.08 ± 13.68	63.03 ± 14.53	166.957	0.000

**Table 5:** Comparison among people with different education level in each dimension of SRHMS (n = 6801)

Dimension	Education				*F*	*P*
			
	Illiterate or other	Low education	Medium education	High education		
**B1**	57.20 ± 16.15	65.77 ± 15.91	68.19 ± 14.51	67.21 ± 14.20	75.447	0.000
**B2**	83.16 ± 19.52	90.71 ± 15.38	94.49 ± 10.82	94.04 ± 12.20	113.243	0.000
**B3**	66.59 ± 24.89	80.56 ± 21.12	88.22 ± 13.93	89.21 ± 14.76	277.202	0.000
**BZT**	67.60 ± 16.26	77.46 ± 14.53	81.82 ± 10.36	81.57 ± 10.81	210.172	0.000
**M1**	68.04 ± 19.46	76.71 ± 17.14	78.33 ± 15.39	79.08 ± 14.92	65.605	0.000
**M2**	65.02 ± 19.66	67.82 ± 20.40	65.61 ± 20.81	65.99 ± 20.02	5.715	0.001
**M3**	53.29 ± 19.64	65.99 ± 19.17	71.81 ± 16.62	73.70 ± 16.04	215.455	0.000
**MZT**	63.68 ± 15.17	70.41 ± 15.19	71.09 ± 14.30	71.89 ± 14.22	42.838	0.000
**S1**	63.35 ± 17.42	75.07 ± 16.19	79.77 ± 14.00	80.93 ± 13.67	216.121	0.000
**S2**	58.55 ± 21.62	69.28 ± 19.83	74.43 ± 17.88	76.13 ± 17.44	142.799	0.000
**S3**	60.41 ± 21.39	65.01 ± 20.42	66.85 ± 19.20	68.37 ± 18.50	25.422	0.000
**SZT**	60.62 ± 16.68	70.14 ± 15.83	74.31 ± 13.91	75.79 ± 13.64	167.929	0.000
**ZCZT**	64.36 ± 13.36	73.06 ± 12.84	76.11 ± 10.44	76.69 ± 10.48	175.703	0.000

**Table 6:** Comparison among different marital status people in each dimension of SRHMS (n = 6801)

Dimension	Marital Status			*F*	*P*
			
	Non-married	Married	Divorced/separated/widowed		
**B1**	70.69 ± 13.79	65.71 ± 15.10	56.48 ± 15.88	200.886	0.000
**B2**	95.28 ± 10.59	92.69 ± 12.80	79.32 ± 22.56	308.725	0.000
**B3**	93.10 ± 10.75	83.35 ± 18.21	64.42 ± 25.91	586.624	0.000
**BZT**	84.51 ± 8.88	78.84 ± 12.47	65.53 ± 17.71	515.427	0.000
**M1**	78.61 ± 14.91	77.82 ± 16.16	67.70 ± 19.13	108.492	0.000
**M2**	65.77 ± 19.23	67.32 ± 20.71	60.74 ± 19.87	27.401	0.000
**M3**	73.27 ± 15.95	68.73 ± 18.35	56.69 ± 20.69	181.154	0.000
**MZT**	71.55 ± 13.78	71.10 ± 14.68	62.25 ± 15.50	99.975	0.000
**S1**	79.80 ± 13.34	77.80 ± 15.37	64.70 ± 18.26	222.157	0.000
**S2**	75.17 ± 18.07	72.07 ± 18.78	60.18 ± 21.93	134.859	0.000
**S3**	67.23 ± 18.90	66.57 ± 19.69	59.88 ± 20.49	32.858	0.000
**SZT**	74.73 ± 13.69	72.61 ± 14.89	61.61 ± 17.46	171.259	0.000
**ZCZT**	77.42 ± 9.76	74.50 ± 11.62	63.35 ± 14.20	325.359	0.000

**Table 7:** Comparison among different cities' residents in each dimension of SRHMS (n = 6801)

Dimension	City					*F*	*P*
			
	Guangzhou	Maoming	Shantou	Shaoguan	Shengzhen		
**B1**	66.29 ± 15.51	66.58 ± 16.01	66.77 ± 15.16	66.46 ± 15.13	65.06 ± 14.68	2.949	0.019
**B2**	92.86 ± 14.42	91.98 ± 13.61	91.91 ± 15.06	91.77 ± 13.62	92.19 ± 13.64	1.214	0.303
**B3**	84.10 ± 19.90	84.04 ± 18.81	83.80 ± 20.20	85.05 ± 17.45	83.85 ± 18.60	0.955	0.431
**BZT**	79.34 ± 13.38	79.18 ± 13.30	79.17 ± 13.91	79.37 ± 12.57	78.56 ± 12.55	0.925	0.448
**M1**	78.05 ± 16.91	78.04 ± 15.55	77.71 ± 15.29	76.37 ± 16.07	76.05 ± 17.89	4.832	0.001
**M2**	67.96 ± 19.50	65.70 ± 20.68	66.29 ± 20.48	66.74 ± 20.86	64.45 ± 21.41	5.665	0.000
**M3**	71.76 ± 17.90	68.63 ± 17.86	69.06 ± 18.93	68.66 ± 17.81	67.07 ± 19.25	12.028	0.000
**MZT**	72.08 ± 14.83	70.40 ± 14.39	70.65 ± 14.34	70.33 ± 14.87	68.84 ± 15.51	8.728	0.000
**S1**	79.29 ± 15.72	77.56 ± 14.64	76.93 ± 16.01	76.62 ± 15.11	76.04 ± 16.57	8.773	0.000
**S2**	75.63 ± 19.15	73.06 ± 18.36	71.08 ± 19.36	71.49 ± 18.03	68.87 ± 20.70	24.302	0.000
**S3**	67.29 ± 21.84	69.63 ± 17.54	64.77 ± 19.44	67.49 ± 18.48	62.52 ± 20.15	28.363	0.000
**SZT**	74.77 ± 15.44	73.71 ± 14.07	71.45 ± 13.34	72.20 ± 14.67	69.67 ± 16.14	24.41	0.000
**ZCZT**	75.62 ± 12.17	74.69 ± 11.73	74.16 ± 11.94	74.33 ± 11.87	72.82 ± 12.22	10.368	0.000

**Table 8:** Comparison among people with different incomes in each dimension of SRHMS (n = 6801)

Dimension	income				*F*	*P*
			
	0~	500~	1000~	2000~		
**B1**	64.82 ± 15.22	66.17 ± 15.53	67.76 ± 14.31	66.24 ± 14.85	9.365	0.000
**B2**	90.36 ± 15.42	91.29 ± 14.73	93.04 ± 12.34	94.23 ± 11.23	20.28	0.000
**B3**	81.08 ± 21.41	83.73 ± 18.13	85.39 ± 17.09	87.62 ± 14.66	29.107	0.000
**BZT**	77.12 ± 14.12	78.73 ± 13.30	80.38 ± 11.67	80.76 ± 10.93	23.379	0.000
**M1**	75.23 ± 17.82	75.79 ± 16.75	78.83 ± 15.46	79.80 ± 15.10	23.782	0.000
**M2**	64.54 ± 20.42	64.62 ± 21.73	66.71 ± 20.51	66.87 ± 20.45	4.913	0.002
**M3**	65.43 ± 18.86	69.75 ± 17.16	71.92 ± 17.05	72.76 ± 17.98	46.182	0.000
**MZT**	68.28 ± 15.05	69.37 ± 15.33	71.79 ± 14.06	72.36 ± 14.79	22.478	0.000
**S1**	73.75 ± 16.48	77.18 ± 15.39	80.42 ± 14.22	80.95 ± 14.46	64.722	0.000
**S2**	68.68 ± 19.75	71.00 ± 18.70	74.52 ± 18.40	75.10 ± 19.36	33.696	0.000
**S3**	64.17 ± 19.57	66.39 ± 20.07	67.79 ± 19.36	66.41 ± 20.33	8.311	0.000
**SZT**	69.25 ± 15.72	71.91 ± 15.25	74.81 ± 14.43	74.88 ± 14.89	43.379	0.000
**ZCZT**	71.96 ± 12.51	73.68 ± 12.34	75.93 ± 10.78	76.29 ± 11.22	40.002	0.000

First, results showed that women had a significant lower SRH scores in all dimension compared with men except for social support (S3, P > 0.05) (Table 3). However, men had the lowest score (65.83 ± 19.79) in social support dimension (S3), while women had the lowest score (64.83 ± 15.23) in physical symptom and organic function dimension (B1).

Table 4 shows that older age groups had lower SRHMS scores in all dimensions except for the positive emotion (M2, P = 0.746). People whose ages range from 25 to 45 had a relative higher scores in every dimension.

Table 5 shows the result of the comparison among people with different education level in each dimension of SRHMS. A strong relationship was observed between education and SRH. People with education overall have significant higher scores in all dimensions than the illiterates. There were also significant differences among different education groups in all dimensions (p < 0.01). People with higher education level generally had higher SRH scores.

A strong relationship between SRH and marital status was also observed in this study. The SRH scores of divorced/separated/widowed group were significantly lower than those of non-married and married group in all dimensions (Table 6). Non-married group had higher scores than married people in all dimensions except for the positive emotion (M2) (P < 0.01).

Participants from Guangzhou had the highest scores in psychosocial health subscale (MZT = 72.08 ± 14.83) and social health subscale (SZT = 74.77 ± 15.44) (Table 7). Scores of physical health subscale (SZT) were not found significant differences among the people of these 5 cities (P = 0.448). In this study we also found that residence location had an effect on SRH status. Generally, people who lived in urban had higher scores in most dimensions (P < 0.05). However, the urban residents showed a lower social support (S3) score than residents in rural, which might be explained by the fact that rural residents have a closer relationship with their neighbors and relatives.

Finally, table 8 shows that economic status had a great influence on the SRH status and high income was an important factor for a good SRH status. People with higher incomes had significantly higher SRHMS scores than poor residents (P < 0.01).

## Discussion

Patient-orientation and empowerment have become important goals for primary care providers[25]. SRH is one of the most important patient-oriented outcomes, and therefore is an appropriate focus for managerial research in primary care[25]. Although the single item SRH was concerned to be enough to reveal people's health status, it can't give more specific information about the health status. One reason is that managers or policy makers are unlikely to be willing to make significant management or policy decisions based on data from a single item[28]. Furthermore, specific actions are unlikely to be suggested by data from a single item for managers to consider. Health status is too complex to be addressed by single items. Our research is based on the definition of health by WHO, which conforms to the transition of the health measurement from the single dimension to the multiple dimensions, from group to individual, from negative to positive and from object to subject. We referred to the internationally and nationally applied scales of psychology and sociology, integrated the Chinese culture, social structure and concept of value, and absorbed the latest achievements of the humanities to thoughtfully consider the cognition and expectation of the individual SRH. The indices were screened from the physical, mental and social health to completely and accurately reflect the contents of SRH. We constructed the method on quantitative measurement of SRH and developed the SRH measurement scale (SRHMS).

Previous studies[29-35] have shown that: (1) SRHMS is reliable, valid and sensitive. (2) SRHMS can show the real connotation of SRH conception quantitatively, correctly, totally and definitely. (3)SRHMS is economic, easy to be used, and adaptable in China. In this study, we explored the performances of the revision of SRHMS in large scale population groups in Southern China.

In this study, the performance of the questionnaire in large scale population survey was satisfactory. We found that women had significantly poorer SRH in all dimensions compared with men, but for the dimension of social support. Other available studies also obtained the similar result[36]. The results were interesting, because women on average live longer than men in China[37]. This phenomenon can be interpreted as gender inequalities in health in China. Although effective measures had been adopted to improve the gender equalities these years, women still have less access to information, education, employment or other social affairs[38,39]. Today most women in China have to go out for work, but they still have to do most of the housework and care for their children[40]. All the above facts definitely generate a worse health status for women. Similar results were also found in other Chinese population[36,41,42]. Although women on average live longer than men, they are reported more illness than men. Estimates of healthy life expectancy from *World health report 2002 *also showed that in almost all countries women have fewer healthy years of the life than men[43].

As other available studies shown[17,18,25,41], ages has a negative effect on the health status. With the increasing age the average scores of SRHMS decrease. However, it seems that there was not significant difference among people with different age in psychosocial symptom (M2). That result may suggest that age negatively affects the SRH status mostly on physical health than mental health. This issue deserves further research because it may have important implications in health services decision making based on this health indicator.

A strong association was observed between education and SRH. People with higher education level appear overall to be healthier than people with less education. The result was similar to some available studies[44,45]. According to Ross and Wu[46], the positive association between education and health can be explained by that high educational attainment improves health directly, and it improves health indirectly through work and economic conditions, social-psychological resources, and health lifestyle. Comparing with the poorly educated, well educated respondents are more likely to have fulfilling job and high income. They obtain more health knowledge and have a greater sense of controlling their behaviors, of living a health lifestyle. But to examine whether the association between education and SRH is true, the result should minimize the influence of the age. In China, most of the elders have less education than the youths, because there was only a little chance for people to get formal education in the past. Age is a negative factor for the health. Therefore, to reveal the relation between education and health status, the influence of age should be eliminate.

Like the education, the present study also showed a strong association between marital and the SRH status. In China unmarried cohabitation is still not widely accepted due to the moral, social, and historical views. As the values and cultural backgrounds are different, unmarried cohabitation is much rarer in China than that in European. Marital status in this survey refers only to legal marital status. We also found that among all the samples very few people are unmarried cohabitation. Therefore, we classified there people in the unmarried group. As the result shown, non-married people enjoyed a higher SRH status than married, widowed, single and divorced persons. Similar results were found in other studies[41,42,44]. Non-married people have higher scores than married people in all dimensions except the Positive emotion dimension (M2). This phenomenon may be explained by the fact that most of the non-married participants are young people. They have good physical health, but are lack of mature emotion. Similarly lack of emotional support, practical support and feelings of loneliness may be the reasons for the poor SRH status of the widowed, single and divorced persons.

The present study also strongly supports our hypothesis that people who live in the urban district have good SRH status. That may contribute to the convenience of the health care, education, entertainment and so on in city. But in the dimension of social support, the urban residents' SRH statuses were poorer than the rural residents'. In China, especially in Southern China, rural residents have a close relation with their neighbors. Even in some villages, all the villagers have the same family name. Most of them originated from one family hundreds years ago. They can often get supports from each other. But it seldom appears in city. Most people, who live in the urban district, even don't know who are their next-door after several years. This may be the explanation that urban residents' social supports were poorer than rural residents'.

Last but not the least, economic status also has a great influence on the SRH status[44,45]. High incomes are important for SRH status. Enough income provides residents more chances for health care and entertainment. Although the health care system has been reformed, it's still hard for people with low income to get the health care they need. That is one of the reasons that poor residents have lower SRHMS scores compared with rich residents.

The clear gradient of SHRMS scores observed according to SRH status in different population groups supports the discriminated validity of the instrument. High complete rate and the low percentage of missed data also show the satisfactory performances of SHRMS.

In this study, the performance of the questionnaire in large scale survey is satisfactory and provides a large picture of SRH status in Southern China. However, several limitations need to be taken into account when interpreting our findings. Firstly, the study design was cross-sectional and it is hence difficult to establish cause-effect relationship between SRH and socio-demographic characteristics. A longitudinal study is needed to investigate the relationship in the future study. Secondly, previous studies have indicated an association of the medical conditions (multimorbidity) with a poor SRH[47-49], suggesting the existence of an inverse relationship between multimorbidity and SRH. Particularly, the inverse relationship between multimorbidity and SRH is significantly stronger in physical dimensions than in mental or social dimension[47-49]. However, in this study we didn't take the multimorbidity and medical condition into consideration. Thirdly, the scale included 48 items and it's difficult for some responders with poor education to understand. Although all the interviewers received uniform training and investigation were conducted based on the requirement of stratified random sampling, during the investigation interviewers' explanation or introduction might still affect the survey results. Fourthly, there are a number of socio-demographic factors are not included in the survey, such as insurance, living arrangement, family ties, relationship and support, childlessness and number of children. Lastly, floating population is not included in the survey. Guangzhou and Shenzhen are the two biggest metropolises in Southern China that have a large proportion of floating population. In our study we didn't include the floating population in the survey because the selection of the subject is based on the eligible candidates listed in the residential registration record and it's difficult to get the information of floating population. Despite these limitations, the results of this analysis provide a large picture of the SRH status in Southern China, which may facilitate further investigation by using a prospective study design.

## Conclusions

Many experts believe that the 21st century health care system should provide respectful and responsive health care according to individual patient preferences, needs, and values, and ensure that patient values guide all clinical decisions[50]. Patient-orientation and empowerment have become important goals for primary health care providers. As SRH largely determines the need of health services and greatly influences people's activities and their life-satisfaction, validity and reliability scales are needed to be developed. SRHMS has been shown a suitable indicator for the general population health. Our previous studies have revealed that SRHMS has a good validity, reliability and is easy to manage. The results of the present study have also shown that SRHMS displays a good performance in general population. In this study, we found that divorced/separated/widowed people, low income people, low education level, old men, women and suburban residents in Southern China suffered a relatively poor SRH. To improve people's SRH in Southern China, more attention should be paid to and more work should be done for these people. For instance, public health surveillance system can be established to monitor the prevalence of health risk behaviors. SRH assessments are valid health status indicators and can be used in studies and population health monitoring. In many developed countries, public health surveillance system has been established and used as valid measures of health in epidemiologic research and in population health monitoring. China can also learn from these systems to improve population health. To improve country's health service, executive powers in the nation's regional health service should be decentralized. Regulations and guidelines intended to improved health care should be implemented more sufficiently. More effort should be make to strengthen health services for the special population and the needy, which include mothers, children, the elderly, the disabled, students, and those suffering from chronic diseases. Local public health should pay more attention to the mental health of population, especially in urban white-collar crowd. Fast life style, pressure from work and lack of social relations and support all result in poor mental health. Therefore, it's necessary to provide appropriate health service such as periodical psychological consulting and stress relief for this special population.

The result of this study may help reveal the population health level, evaluate the health service effectiveness and provide new method for the formulation of the public health strategies. But more information need to be collected before new public health strategies being using in the primary health care in the future.

## Competing interests

The authors declare that they have no competing interests.

## Authors' contributions

JX developed the questionnaire and study design, supervised the analysis and contributed to the final version of the manuscript. JZ who assisted with the survey and data analyses is also the principal author of this paper. LF, JQ assisted with the study. All authors have read and approved the final manuscript.

## Funding

This research was supported by the National Natural Science Foundation of China (Methodological Study on Quantitative Measurement of Self-rated Health. NO: 30100154)

## Pre-publication history

The pre-publication history for this paper can be accessed here:

http://www.biomedcentral.com/1471-2458/10/393/prepub
